# Supplementary material for: Comparative Gene Co-expression Network Analysis of Proviral and Antiviral Responses to Dengue Virus-2 (DENV-2) and Zika Virus (ZIKV) Infection in Human Neural Progenitor Cells (hNPCs)
Source: PLoS One. 2026 Apr 30;21(4):e0347540. doi: 10.1371/journal.pone.0347540 (PMC13132211; doi:10.1371/journal.pone.0347540)
Supplement: S1 Fig — (DOCX) [file pone.0347540.s002.docx]

**S1_Fig. Principal component analysis (PCA) of the 500 most variable genes.**

|  |
| --- |
