# Supplementary figures and images for: Comparative Gene Co-expression Network Analysis of Proviral and Antiviral Responses to Dengue Virus-2 (DENV-2) and Zika Virus (ZIKV) Infection in Human Neural Progenitor Cells (hNPCs)
Source: PLoS One. 2026 Apr 30;21(4):e0347540. doi: 10.1371/journal.pone.0347540 (PMC13132211; doi:10.1371/journal.pone.0347540)

**S2_Fig. The optimal soft-thresholding power (β) for constructing an unsigned scale-free network.**

| 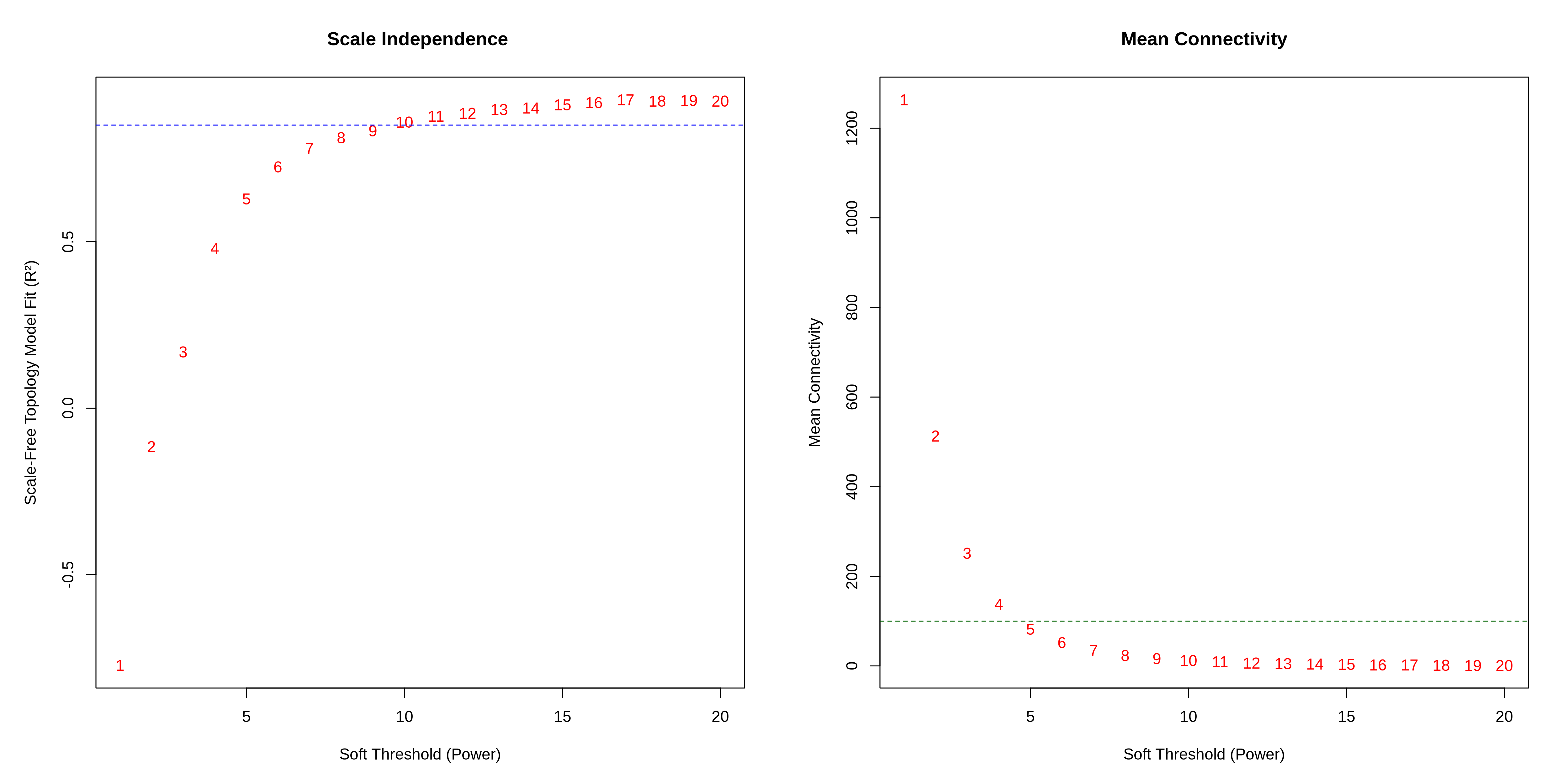 |
| --- |

Supplement: S2 Fig — (DOCX) [file pone.0347540.s003.docx]
