# Supplementary material for: Comparative Gene Co-expression Network Analysis of Proviral and Antiviral Responses to Dengue Virus-2 (DENV-2) and Zika Virus (ZIKV) Infection in Human Neural Progenitor Cells (hNPCs)
Source: PLoS One. 2026 Apr 30;21(4):e0347540. doi: 10.1371/journal.pone.0347540 (PMC13132211; doi:10.1371/journal.pone.0347540)
Supplement: S1 File — (DOCX) [file pone.0347540.s007.docx]

**S1_Code. All author-generated code.**

1. WGCNA network construction

IN R

C1

# === Load required packages ===

library(DESeq2)

library(edgeR)

library(WGCNA)

library(pcaExplorer)

library(dynamicTreeCut)

library(clusterProfiler)

library(markdown)

# Load vst-normalized, batch-corrected counts

counts <- read.csv("vst_combat_corrected.csv", row.names = 1)

# Load metadata

metadata <- read.csv("metadata.csv", row.names = 1)

# Convert counts to matrix

vst_mat <- as.matrix(counts)

# Transpose for WGCNA (samples = rows, genes = columns)

datExpr <- t(vst_mat)

# Load metadata (with row names)

metadata <- read.csv("metadata.csv", row.names = 1)

# Check and clean the expression data

gsg <- goodSamplesGenes(datExpr, verbose = 3)

if (!gsg$allOK) {

datExpr <- datExpr[gsg$goodSamples, gsg$goodGenes]

}

# Report retained samples and genes

cat("Samples kept:", sum(gsg$goodSamples), "\n")

cat("Genes kept:", sum(gsg$goodGenes), "\n")

# === Step 6: Choose soft-thresholding power ===

powers <- c(1:20)

sft <- pickSoftThreshold(datExpr, powerVector = powers, verbose = 5, networkType = "unsigned")

# Plot scale-free topology fit and mean connectivity

par(mfrow = c(1,2))

# Scale-free topology model fit

plot(sft$fitIndices[,1], -sign(sft$fitIndices[,3]) * sft$fitIndices[,2],

xlab = "Soft Threshold (Power)", ylab = "Scale-Free Topology Model Fit (R²)",

type = "n", main = "Scale Independence")

text(sft$fitIndices[,1], -sign(sft$fitIndices[,3]) * sft$fitIndices[,2],

labels = powers, col = "red")

abline(h = 0.85, col = "blue", lty = 2)

# Mean connectivity plot

plot(sft$fitIndices[,1], sft$fitIndices[,5],

xlab = "Soft Threshold (Power)", ylab = "Mean Connectivity",

type = "n", main = "Mean Connectivity")

text(sft$fitIndices[,1], sft$fitIndices[,5], labels = powers, col = "red")

abline(h = 100, col = "darkgreen", lty = 2) # desired threshold

# Print mean connectivity values

connectivity_df <- data.frame(Power = sft$fitIndices[,1],

ScaleFreeFit = sft$fitIndices[,2],

MeanConnectivity = sft$fitIndices[,5])

print(connectivity_df)

# Select a power with R² > 0.80 and Mean Connectivity > 100

candidate_powers <- connectivity_df %>%

filter(ScaleFreeFit > 0.80, MeanConnectivity > 100)

if (nrow(candidate_powers) > 0) {

softPower <- candidate_powers$Power[1] # choose the first matching power

} else {

softPower <- sft$powerEstimate # fallback

}

cat("Chosen soft-thresholding power:", softPower, "\n")

# === Step 7: Build the unsigned co-expression network and detect modules ===

net <- blockwiseModules(datExpr,

power = softPower,

networkType = "unsigned", # changed to unsigned

TOMType = "unsigned", # change TOM type

minModuleSize = 30,

reassignThreshold = 0,

mergeCutHeight = 0.25,

numericLabels = TRUE,

pamRespectsDendro = FALSE,

saveTOMs = TRUE,

saveTOMFileBase = "TOM_block_unsigned",

verbose = 3)

# === Choose soft-thresholding power for unsigned network ===

powers <- c(1:20)

sft <- pickSoftThreshold(datExpr, powerVector = powers, verbose = 5, networkType = "unsigned")

# === Save high-resolution soft-thresholding plots ===

png("soft_threshold_plots_unsigned.png", width = 5000, height = 2500, res = 300)

par(mfrow = c(1,2), mar = c(5,5,4,2) + 0.1)

# Scale-free topology model fit

plot(sft$fitIndices[,1], -sign(sft$fitIndices[,3]) * sft$fitIndices[,2],

xlab = "Soft Threshold (Power)", ylab = "Scale-Free Topology Model Fit (R²)",

type = "n", main = "Scale Independence ")

text(sft$fitIndices[,1], -sign(sft$fitIndices[,3]) * sft$fitIndices[,2],

labels = powers, col = "red")

abline(h = 0.85, col = "blue", lty = 2)

# Mean connectivity plot

plot(sft$fitIndices[,1], sft$fitIndices[,5],

xlab = "Soft Threshold (Power)", ylab = "Mean Connectivity",

type = "n", main = "Mean Connectivity")

text(sft$fitIndices[,1], sft$fitIndices[,5], labels = powers, col = "red")

abline(h = 100, col = "darkgreen", lty = 2)

dev.off()

# Step 8: Draw dendrogram and module colors for All Genes

# Convert numeric labels to colors

moduleColors <- labels2colors(net$colors)

# Extract gene indices and dendrogram for the only block (contains all genes)

all_genes <- net$blockGenes[[1]]

dend_all <- net$dendrograms[[1]]

# Extract module colors for all genes

moduleColors_all <- moduleColors[all_genes]

# Optional: Check lengths before plotting

dend_labels <- labels(as.dendrogram(dend_all))

cat("Length of module colors:", length(moduleColors_all), "\n")

cat("Number of genes in dendrogram:", length(dend_labels), "\n")

if (length(moduleColors_all) != length(dend_labels)) {

stop("❌ Length mismatch: Number of module colors ≠ dendrogram leaves. Check your data!")

}

# Plot dendrogram with module colors

plotDendroAndColors(

dendro = dend_all,

colors = moduleColors_all,

groupLabels = "Module Colors",

dendroLabels = FALSE,

hang = 0.03,

main = "Gene Cluster Dendrogram"

)

# === Save dendrogram with high resolution (1500 DPI) ===

png(filename = "Dendrogram_ModuleColors_AllGenes.png",

width = 10, height = 8, units = "in", res = 1500, type = "cairo")

# Plot dendrogram

plotDendroAndColors(

dendro = dend_all,

colors = moduleColors_all,

groupLabels = "Module Colors",

dendroLabels = FALSE,

hang = 0.03,

main = "Gene Cluster Dendrogram"

)

dev.off()

2. Correlation Heatmap

C2

IN R

# ==== Load Required Libraries ====

library(WGCNA)

library(ComplexHeatmap)

library(circlize)

library(grid)

options(stringsAsFactors = FALSE)

# ==== Load and Prepare Expression Data ====

expr_data <- read.csv("vst_combat_corrected.csv", row.names = 1)

datExpr <- t(expr_data)# ==== Load Required Libraries ====

library(WGCNA)

library(ComplexHeatmap)

library(circlize)

library(grid)

options(stringsAsFactors = FALSE)

# ==== Load and Prepare Expression Data ====

expr_data <- read.csv("vst_counts.csv", row.names = 1)

datExpr <- t(expr_data)

# Ensure numeric matrix

datExpr_numeric <- apply(datExpr, 2, as.numeric)

rownames(datExpr_numeric) <- rownames(datExpr)

colnames(datExpr_numeric) <- colnames(datExpr)

# ==== Define Sample Names for 16 Samples ====

sample_names <- c(paste0("Dengue", 1:15), paste0("Zika", 1:15))

rownames(datExpr) <- sample_names

# Check dimensions

print(dim(datExpr_numeric)) # should be 30 samples x genes

# If not, transpose

if (nrow(datExpr_numeric) != 30) {

datExpr_numeric <- t(datExpr_numeric)

}

# ==== Build WGCNA Network ====

softPower <- 4

net <- blockwiseModules(

datExpr_numeric,

power = softPower,

TOMType = "unsigned",

minModuleSize = 30,

reassignThreshold = 0,

mergeCutHeight = 0.25,

numericLabels = TRUE,

pamRespectsDendro = FALSE,

saveTOMs = FALSE,

verbose = 3

)

# ==== Assign Colors to Modules ====

moduleColors <- labels2colors(net$colors)

table(moduleColors)

# ==== Calculate MEs (Module Eigengenes) ====

MEs <- moduleEigengenes(datExpr, colors = moduleColors)$eigengenes

MEs <- orderMEs(MEs)

# ==== Rename ME columns to color names ====

me_names <- gsub("^ME", "", colnames(MEs))

colnames(MEs) <- me_names

rownames(MEs) <- sample_names

# ==== Create Group Trait Matrix with Full Labels ====

group_traits <- data.frame(

"Infected with DENV2" = grepl("^Dengue", sample_names),

"Infected with ZIKV" = grepl("^Zika", sample_names),

check.names = FALSE # Prevents conversion of spaces to dots

)

rownames(group_traits) <- sample_names

# ==== Correlation: MEs vs. Group Traits ====

moduleGroupCor <- cor(MEs, group_traits, use = "p")

moduleGroupPval <- corPvalueStudent(moduleGroupCor, nSamples = nrow(datExpr))

moduleGroupPval_adj <- t(apply(moduleGroupPval, 1, p.adjust, method = "fdr"))

# ==== Set row names ====

rownames(moduleGroupCor) <- me_names

# ==== Create Row Annotation for Heatmap ====

row_ha <- rowAnnotation(

Module = anno_simple(

x = me_names,

col = setNames(me_names, me_names),

border = TRUE,

pch = NA,

gp = gpar(col = NA)

),

annotation_name_side = "top",

annotation_width = unit(5, "mm")

)

# ==== Plot Heatmap: Module vs. Group Correlation ====

Heatmap(

moduleGroupCor,

name = "Correlation",

col = colorRamp2(c(-1, 0, 1), c("blue", "white", "red")),

cluster_rows = FALSE,

cluster_columns = FALSE,

column_title = "Module_Triat relationship",

left_annotation = row_ha,

row_names_side = "left",

row_names_gp = gpar(fontsize = 12),

column_names_gp = gpar(fontsize = 12),

show_row_names = TRUE,

show_column_names = TRUE,

cell_fun = function(j, i, x, y, width, height, fill) {

grid.text(

sprintf("%.2f\n%s", moduleGroupCor[i, j], signif(moduleGroupPval_adj[i, j], 2)),

x, y, gp = gpar(fontsize = 10)

)

}

)

# Save the heatmap as a PNG file with 1500 dpi

png("Module_Trait_Correlation_Heatmap.png",

width = 10, height = 8, units = "in", res = 1500)

# Re-plot the heatmap inside the PNG device

Heatmap(

moduleGroupCor,

name = "Correlation",

col = colorRamp2(c(-1, 0, 1), c("blue", "white", "red")),

cluster_rows = FALSE,

cluster_columns = FALSE,

column_title = "Module–Trait Relationships",

left_annotation = row_ha,

row_names_side = "left",

row_names_gp = gpar(fontsize = 12),

column_names_gp = gpar(fontsize = 12),

show_row_names = TRUE,

show_column_names = TRUE,

cell_fun = function(j, i, x, y, width, height, fill) {

grid.text(

sprintf("%.2f\np=%.2g", moduleGroupCor[i, j], moduleGroupPval_adj[i, j]),

x, y, gp = gpar(fontsize = 10)

)

}

)

dev.off() # Important to close and save the file

3. Hub gene identification

IN R

C3

# Identify hub genes in each non-grey module

hubs <- chooseTopHubInEachModule(datExpr_numeric, moduleColors)

# Build dataframe

hub_df <- data.frame(Module = names(hubs), HubGene = hubs)

# Save

write.csv(hub_df, "hub_genes.csv", row.names = FALSE)

library(biomaRt)

# Connect to Ensembl (human)

ensembl <- useMart("ensembl", dataset = "hsapiens_gene_ensembl")

# New hub genes from your list

hub_genes <- c(

"ENSG00000221983",

"ENSG00000151422",

"ENSG00000198898",

"ENSG00000112972",

"ENSG00000157796",

"ENSG00000171951",

"ENSG00000167191",

"ENSG00000162490",

"ENSG00000139668",

"ENSG00000179218",

"ENSG00000105778"

)

# Get data from biomaRt

hub_annotation_raw <- getBM(

attributes = c(

"ensembl_gene_id",

"external_gene_name",

"ensembl_peptide_id",

"description"

),

filters = "ensembl_gene_id",

values = hub_genes,

mart = ensembl

)

library(dplyr)

hub_annotation_summary <- hub_annotation_raw %>%

group_by(ensembl_gene_id, external_gene_name, description) %>%

summarise(

ensembl_peptide_id = paste(unique(ensembl_peptide_id[ensembl_peptide_id != ""]), collapse = ", ")

) %>%

ungroup()

# View the summary table (one row per gene)

print(hub_annotation_summary)

# Save to CSV if you want

write.csv(hub_annotation_summary, "hub_genes_protein_summary.csv", row.names = FALSE)

4. Module Membership between Gene significance

In R

C4

# ==== Get trait expression profile for "Infected with ZIKV" ====

infected_ZIKV_expr <- group_traits[,"Infected with ZIKV"] # binary vector: 1 = infected, 0 = not

# ==== Calculate Module Membership (MM) as usual ====

MM <- cor(datExpr, MEs, use = "p") # all genes × all modules

MM_pval <- corPvalueStudent(MM, nSamples = nrow(datExpr))

# ==== Calculate Gene Significance (GS) for the trait "Infected with ZIKV" ====

GS <- cor(datExpr, infected_ZIKV_expr, use = "p")

GS_pval <- corPvalueStudent(GS, nSamples = nrow(datExpr))

# ==== Subset for green module genes ====

green_genes <- moduleColors == "green"

# ==== Compute correlation between MM and GS for green module ====

mm_green <- abs(MM[green_genes, "green"])

gs_green <- abs(GS[green_genes])

cor_result <- cor.test(mm_green, gs_green, method = "pearson")

cor_r <- round(cor_result$estimate, 6)

cor_p <- signif(cor_result$p.value, 6)

# ==== Save plot to PNG at 1500x1500 resolution ====

png("green_module_MM_vs_GS_zika.png", width = 1500, height = 1500, res = 300)

# ==== Plot MM vs GS for green module genes ====

plot(

mm_green,

gs_green,

xlab = "Module Membership in Green Module",

ylab = "Gene Significance for Infected with ZIKV",

main = paste0("Green Module: MM vs GS\nr = ", cor_r, ", p = ", cor_p),

pch = 19,

col = "darkgreen"

)

dev.off()

5. Draw Gene co-expression network plot

In R

C5

# ==== Required libraries ====

library(WGCNA)

library(igraph)

# 1. Identify the turquoise module hub gene

turquoise_genes <- (moduleColors == "turquoise")

hub_turquoise <- chooseTopHubInEachModule(datExpr_numeric, moduleColors)["turquoise"]

cat("Turquoise hub gene:", hub_turquoise, "\n")

# 2. Get TOM similarity for turquoise genes

TOM <- TOMsimilarityFromExpr(datExpr_numeric, power = softPower)

turquoise_index <- which(moduleColors == "turquoise")

turquoise_TOM <- TOM[turquoise_index, turquoise_index]

# 3. Convert to adjacency-like matrix for graphing

dimnames(turquoise_TOM) <- list(colnames(datExpr_numeric)[turquoise_index],

colnames(datExpr_numeric)[turquoise_index])

# 4. Subset to top connections for the hub gene

hub_gene <- hub_turquoise

hub_connections <- sort(turquoise_TOM[hub_gene, ], decreasing = TRUE)

# 5. Keep top 30 connections (you can adjust)

top_hub_connections <- names(hub_connections)[1:30]

subTOM <- turquoise_TOM[top_hub_connections, top_hub_connections]

#6. Build igraph object (using new function)

g <- graph_from_adjacency_matrix(

subTOM,

mode = "undirected",

weighted = TRUE,

diag = FALSE

)

# 7. Node colors: hub gene in bright orange, others in bright cyan

vertex_colors <- rep("cyan", vcount(g)) # other nodes

vertex_colors[which(V(g)$name == hub_gene)] <- "orange" # hub gene

# 8. Node sizes: proportional to degree (number of connections)

vertex_sizes <- degree(g)

vertex_sizes <- 5 + 3 * (vertex_sizes / max(vertex_sizes)) # scale sizes

# 9. Edge widths: proportional to weight (co-expression strength)

edge_widths <- E(g)$weight * 5 # adjust multiplier for visibility

# 10. Plot network with increased resolution and larger gene labels

png("turquoise_hub_network.png", width = 1500, height = 1500, res = 150)

par(mar = c(1, 1, 1, 1))

plot(

g,

vertex.size = vertex_sizes,

vertex.label.cex = 1.0,, # increase label size for clarity

vertex.color = vertex_colors,

edge.width = edge_widths,

edge.color = "gray50",

main = paste("Hub gene network – Turquoise module (Infected with DENV2)")

)

dev.off()

6. Donut chart for module inclduong the grey module

In R

C6

# ==== Load Required Libraries ====

library(ggplot2)

library(dplyr)

library(WGCNA)

# ==== Recalculate or Convert Module Labels to Colors ====

# This assumes you already have WGCNA results loaded as 'net'

moduleColors <- labels2colors(net$colors) # Convert numeric labels to color names

# ==== Create Module Counts Table ====

module_counts <- as.data.frame(table(moduleColors)) # Count genes per module

# ==== Add Label for Plotting (only gene counts shown) ====

module_counts <- module_counts %>%

mutate(Label = Freq)

# ==== Create Donut Chart and Save as PNG ====

# Open PNG device with high resolution

png("module_donut_chart.png", width = 1500, height = 1500, res = 300)

# Plot

p <- ggplot(module_counts, aes(x = 2, y = Freq, fill = moduleColors)) +

geom_bar(stat = "identity", width = 1, color = "white") +

coord_polar("y", start = 0) +

xlim(0.5, 2.5) +

geom_text(

aes(label = Label),

position = position_stack(vjust = 0.5),

size = 1.5,

color = "white"

) +

scale_fill_manual(values = as.character(module_counts$moduleColors)) +

labs(title = "Gene Count per Module", fill = "Module") +

theme_void() +

theme(

legend.position = "right",

legend.text = element_text(size = 9),

plot.title = element_text(hjust = 0.5, size = 18, face = "bold", margin = margin(b = 20))

)

print(p)

dev.off()

7. Functional GOBP enrichment analysis (clusterProfiler)

In R

C7

PART1

# ==== Load Required Libraries ====

library(WGCNA)

library(clusterProfiler)

library(org.Hs.eg.db)

# ==== Step 1: Prepare Expression and Trait Data ====

# Define sample names (must match the WGCNA input)

sample_names <- c(paste0("Dengue", 1:15), paste0("Zika", 1:15))

# Create trait matrix

group_traits <- data.frame(

"Infected with Dengue" = grepl("^Dengue", sample_names),

"Infected with Zika" = grepl("^Zika", sample_names),

check.names = FALSE # Keep spaces in column names

)

rownames(group_traits) <- sample_names

# Use group_traits as trait_data for correlation

trait_data <- group_traits

# ==== Step 2: Identify and extract genes in the Green module ====

# If you used 'datExpr_numeric' or 'datExpr' earlier, extract gene names from it

gene_names <- colnames(datExpr) # or colnames(datExpr_numeric)

# Assign gene names to moduleColors

names(moduleColors) <- gene_names

# Now extract Greenmodule genes

green_genes <- names(moduleColors[moduleColors == "green"])

# ==== Step 3: Correlate module eigengenes with trait data ====

module_trait_cor <- cor(MEs, trait_data, use = "p")

module_trait_pval <- corPvalueStudent(module_trait_cor, nrow(trait_data))

# ==== Step 4: Check correlation of Green module with "Infected with Dengue" ====

correlation_value <- module_trait_cor["green", "Infected with Dengue"]

p_value <- module_trait_pval["green", "Infected with Dengue"]

cat("Correlation:", correlation_value, "\n")

cat("P-value:", p_value, "\n")

# ==== Step 5: Run GO enrichment if strong correlation (positive or negative) and significant ====

if (abs(correlation_value) > 0.5 && p_value < 0.05) {

cat("Proceeding with GO enrichment for strong correlation...\n")

# Assume green_genes are ENSEMBL IDs

bp <- enrichGO(gene = green_genes,

OrgDb = org.Hs.eg.db,

keyType = "ENSEMBL",

ont = "BP",

pAdjustMethod = "BH",

qvalueCutoff = 0.05,

readable = TRUE)

cc <- enrichGO(

gene = green_genes,

OrgDb = org.Hs.eg.db,

keyType = "ENSEMBL",

ont = "CC",

pAdjustMethod = "BH",

qvalueCutoff = 0.05,

readable = TRUE

)

mf <- enrichGO(gene = green_genes,

OrgDb = org.Hs.eg.db,

keyType = "ENSEMBL",

ont = "MF",

pAdjustMethod = "BH",

qvalueCutoff = 0.05,

readable = TRUE)

# Convert to data frames and label ontology

bp_df <- as.data.frame(bp); bp_df$Ontology <- "BP"

cc_df <- as.data.frame(cc); cc_df$Ontology <- "CC"

mf_df <- as.data.frame(mf); mf_df$Ontology <- "MF"

# Combine all and sort

go_combined <- rbind(bp_df, cc_df, mf_df)

go_sorted <- go_combined[order(go_combined$Ontology, go_combined$p.adjust), ]

# Save only one combined CSV file

write.csv(go_sorted, "GO_Combined.csv", row.names = FALSE)

} else {

cat("Green module is not significantly correlated with Infected with Dengue.\n")

}

PART2

library(ggplot2)

library(dplyr)

library(stringr)

#2. Load data

go_data <- read.csv("GO_Combined.csv")

#3. List of all selected terms (BP + CC + MF)

selected_terms <- c(

# BP terms

"GO:0071560","GO:0071559","GO:0007179","GO:0034976","GO:0016236",

"GO:0006986","GO:0017015","GO:0007265","GO:1903844","GO:0007266",

"GO:1901796","GO:0051056","GO:0007178","GO:0030512","GO:0042542",

"GO:0043123","GO:0045191","GO:0002562","GO:0043122","GO:0016447",

"GO:0002200",

# CC terms

"GO:0070160","GO:0010494","GO:0045121","GO:0033178","GO:0005776",

"GO:0005912","GO:0000421","GO:0000151","GO:0034663",

# MF terms

"GO:0031072","GO:0032561","GO:0031625","GO:0044389","GO:0051117",

"GO:0051082","GO:0019887"

)

#4. Filter only tlibrary(ggplot2)

library(dplyr)

library(stringr)

#2. Load data

go_data <- read.csv("GO_Combined.csv")

#3. List of all selected terms (BP + CC + MF)

selected_terms <- c(

# BP terms

"GO:0071560","GO:0071559","GO:0007179","GO:0034976","GO:0016236",

"GO:0006986","GO:0017015","GO:0007265","GO:1903844","GO:0007266",

"GO:1901796","GO:0051056","GO:0007178","GO:0030512","GO:0042542",

"GO:0043123","GO:0045191","GO:0002562","GO:0043122","GO:0016447",

"GO:0002200",

# CC terms

"GO:0070160","GO:0010494","GO:0045121","GO:0033178","GO:0005776",

"GO:0005912"he selected terms

top_go <- go_data %>% filter(ID %in% selected_terms)

# 5.Wrap long descriptions

top_go <- top_go %>%

mutate(Description_wrap = str_wrap(Description, width = 50))

#6. Convert GeneRatio to numeric

top_go$GeneRatio <- sapply(strsplit(as.character(top_go$GeneRatio), "/"),

function(x) as.numeric(x[1]) / as.numeric(x[2]))

#7.Prepare variables for plotting (using adjusted p-value)

top_go <- top_go %>%

mutate(

negLogPadj = -log10(p.adjust), # use p.adjust

Ontology = factor(Ontology, levels = c("BP", "CC", "MF")),

Description_wrap = factor(Description_wrap, levels = rev(Description_wrap))

)

#8. Plot

png("GO_GreenModule_ZIka_Antiviral_Immune.png", width = 12, height = 10, units = "in", res = 1500)

ggplot(top_go, aes(x = GeneRatio, y = Description_wrap, shape = Ontology)) +

geom_point(aes(size = Count, color = negLogPadj)) +

scale_color_gradient(low = "blue", high = "red", name = "-log10(adj p-value)") +

scale_size(range = c(4, 8), name = "Gene Count") +

scale_shape_manual(

name = "Ontology",

values = c(16, 17, 15),

labels = c("Biological Process (BP)", "Cellular Component (CC)", "Molecular Function (MF)")

) +

labs(

title = "GO Terms – Turquoise Module (Infected with DENV2): Antiviral and Immune Response",

x = "Gene Ratio",

y = "GO Term Description"

) +

theme_bw() +

theme(

axis.text.y = element_text(size = 9),

plot.title = element_text(hjust = 0.5, size = 14, face = "bold")

)

dev.off()

Or command for GREEN (INFECTD ZIKV)

library(ggplot2)

library(dplyr)

library(stringr)

# 1. Load data

go_data <- read.csv("GO_Combined.csv") # make sure this file contains all GO terms

# 2. List of selected GO terms for Green module (Infected with ZIKV)

selected_terms <- c(

# BP terms

"GO:0048524","GO:0019079","GO:0031396","GO:0045070","GO:0044794",

"GO:0050792","GO:0044788","GO:0019058","GO:0001768","GO:0001767",

"GO:0010506","GO:1902041","GO:0001780","GO:0044829","GO:0045069",

"GO:1903900",

# CC terms

"GO:0101002","GO:1904813","GO:0035770","GO:0000932","GO:0016605","GO:0010494",

# MF terms

"GO:0031625","GO:0048027","GO:0044389","GO:0070182","GO:0003725","GO:0140662"

)

# 3. Filter only the selected terms

top_go <- go_data %>% filter(ID %in% selected_terms)

# 4. Wrap long descriptions

top_go <- top_go %>%

mutate(Description_wrap = str_wrap(Description, width = 50))

# 5. Convert GeneRatio to numeric

top_go$GeneRatio <- sapply(strsplit(as.character(top_go$GeneRatio), "/"),

function(x) as.numeric(x[1]) / as.numeric(x[2]))

# 6. Prepare variables for plotting

top_go <- top_go %>%

mutate(

negLogPadj = -log10(p.adjust), # use adjusted p-value

Ontology = factor(Ontology, levels = c("BP", "CC", "MF")),

Description_wrap = factor(Description_wrap, levels = rev(Description_wrap))

)

# 7. Plot and save as high-resolution PNG

png("GO_GreenModule_ZIKV_Antiviral_Immune.png", width = 12, height = 10, units = "in", res = 1500)

ggplot(top_go, aes(x = GeneRatio, y = Description_wrap, shape = Ontology)) +

geom_point(aes(size = Count, color = negLogPadj)) +

scale_color_gradient(low = "blue", high = "red", name = "-log10(adj p-value)") +

scale_size(range = c(4, 8), name = "Gene Count") +

scale_shape_manual(

name = "Ontology",

values = c(16, 17, 15),

labels = c("Biological Process (BP)", "Cellular Component (CC)", "Molecular Function (MF)")

) +

labs(

title = "GO Terms – Green Module (Infected with ZIKV): Antiviral and Immune Response",

x = "Gene Ratio",

y = "GO Term Description"

) +

theme_bw() +

theme(

axis.text.y = element_text(size = 9),

plot.title = element_text(hjust = 0.5, size = 14, face = "bold"),

plot.subtitle = element_text(hjust = 0.5, size = 12)

)

dev.off()

20. KEGG enrichment analysis (clusterProfiler)

Part1

C8

# ==== Load Required Libraries ====

library(WGCNA)

library(clusterProfiler)

library(org.Hs.eg.db)

library(DOSE)

# ==== Step 1: Define Sample Names and Trait Data ====

sample_names <- c(paste0("Dengue", 1:15), paste0("Zika", 1:15))

# Create trait matrix (logical TRUE/FALSE)

trait_data <- data.frame(

"Infected with Dengue" = grepl("^Dengue", sample_names),

"Infected with Zika" = grepl("^Zika", sample_names),

check.names = FALSE

)

rownames(trait_data) <- sample_names

gene_names

# ==== Step 2: Extract Green Module Genes ====

# Ensure gene names are set correctly

gene_names <- colnames(datExpr)

names(moduleColors) <- gene_names

# Get green module genes

green_genes <- names(moduleColors[moduleColors == "green"])

# ==== Step 3: Correlate Module Eigengenes with Trait Data ====

module_trait_cor <- cor(MEs, trait_data, use = "p")

module_trait_pval <- corPvalueStudent(module_trait_cor, nrow(trait_data))

# Extract correlation and p-value for Green module vs Dengue

correlation_value <- module_trait_cor["green", "Infected with Dengue"]

p_value <- module_trait_pval["green", "Infected with Dengue"]

cat("Correlation with Dengue (Green module):", correlation_value, "\n")

cat("P-value:", p_value, "\n")

# ==== Step 4: Run KEGG Enrichment if Correlation is Strong and Significant ====

if (abs(correlation_value) > 0.5 && p_value < 0.05) {

cat("Proceeding with KEGG enrichment...\n")

# ==== Step 4.1: Convert Gene IDs ====

# Check if gene IDs are SYMBOL or ENSEMBL

entrez_ids <- tryCatch({

bitr(green_genes,

fromType = "ENSEMBL", # <-- change to "SYMBOL" if needed

toType = "ENTREZID",

OrgDb = org.Hs.eg.db)

}, error = function(e) {

stop("ID conversion failed. Check if green_genes are ENSEMBL or SYMBOL.")

})

# ==== Step 4.2: Run KEGG Enrichment ====

kegg <- enrichKEGG(

gene = entrez_ids$ENTREZID,

organism = "hsa",

pAdjustMethod = "BH",

qvalueCutoff = 0.05

)

# Check if any KEGG pathways are enriched

if (is.null(kegg) || nrow(as.data.frame(kegg)) == 0) {

cat("No significant KEGG pathways found for green module.\n")

} else {

# ==== Step 4.3: Annotate and Save ====

kegg <- setReadable(kegg, OrgDb = org.Hs.eg.db, keyType = "ENTREZID")

kegg_df <- as.data.frame(kegg)

kegg_df <- kegg_df[order(kegg_df$p.adjust), ]

write.csv(kegg_df, "KEGG_Pathway.csv", row.names = FALSE)

cat("KEGG enrichment results saved to 'KEGG_Pathway.csv'\n")

Part2

# ==== Load Required Libraries ====

library(ggplot2)

library(dplyr)

# ==== Step 1: Manually create dataframe with selected KEGG terms ====

kegg_selected <- data.frame(

Pathway = c("Coronavirus disease - COVID-19",

"Systemic lupus erythematosus",

"**Prion disease**",

"Hippo signaling pathway",

"Neutrophil extracellular trap formation",

"Human papillomavirus infection",

"Endocytosis",

"Viral carcinogenesis",

"Phagosome",

"Autophagy - animal",

"PI3K-Akt signaling pathway"),

GeneRatio = c("32/496","32/496","20/496","20/496","23/496","33/496",

"27/496","22/496","18/496","18/496","31/496"),

BgRatio = c("238/9434","278/9434","144/9434","157/9434","196/9434","333/9434",

"252/9434","205/9434","159/9434","169/9434","362/9434"),

pvalue = c(8.29e-07,2.29e-05,6.09e-05,2.05e-04,2.43e-04,3.20e-04,

3.28e-04,1.12e-03,1.70e-03,3.33e-03,4.91e-03),

padjust = c(4.91e-05,0.00076,0.00150,0.00357,0.00376,0.00422,

0.00422,0.01143,0.01602,0.02668,0.03547),

Count = c(32,32,20,20,23,33,27,22,18,18,31)

)

# ==== Step 2: Convert GeneRatio to numeric ====

kegg_selected$GeneRatio <- sapply(strsplit(as.character(kegg_selected$GeneRatio), "/"),

function(x) as.numeric(x[1]) / as.numeric(x[2]))

# ==== Step 3: Order pathways by adjusted p-value ====

kegg_selected <- kegg_selected %>%

arrange(padjust)

kegg_selected$Pathway <- factor(kegg_selected$Pathway,

levels = rev(kegg_selected$Pathway))

# ==== Step 4: Plot barplot (based on adjusted p-value) ====

png("KEGG_Selected_Barplot_padj.png", width = 10, height = 8, units = "in", res = 1500)

ggplot(kegg_selected, aes(x = GeneRatio, y = Pathway)) +

geom_bar(stat = "identity", aes(fill = -log10(padjust)), width = 0.6) + # narrower bars

scale_fill_gradient(low = "blue", high = "red", name = "-log10(adj p-value)") +

labs(title = "KEGG Pathways – Green Module (Infected with Zika): Antiviral and Immune Response",

x = "Gene Ratio",

y = "KEGG Pathway Description") +

theme_bw() +

theme(axis.text.y = element_text(size = 10),

plot.title = element_text(hjust = 0.5, size = 14, face = "bold"))

dev.off()

9. Reactome enrichment analysis (clusterProfiler)

**C9**

Part1

# Check i# ==== Load Required Libraries ====

library(WGCNA)

library(clusterProfiler)

library(org.Hs.eg.db)

library(ReactomePA)

library(DOSE)

# ==== Step 1: Define Sample Names and Trait Data ====

sample_names <- c(paste0("Dengue", 1:15), paste0("Zika", 1:15))

# Create trait matrix (logical TRUE/FALSE)

trait_data <- data.frame(

"Infected with Dengue" = grepl("^Dengue", sample_names),

"Infected with Zika" = grepl("^Zika", sample_names),

check.names = FALSE

)

rownames(trait_data) <- sample_names

# ==== Step 2: Extract Green Module Genes ====

gene_names <- colnames(datExpr) # Ensure gene names match expression data

names(moduleColors) <- gene_names # Link module colors to genes

green_genes <- names(moduleColors[moduleColors == "green"])

# ==== Step 3: Correlate Module Eigengenes with Trait Data ====

module_trait_cor <- cor(MEs, trait_data, use = "p")

module_trait_pval <- corPvalueStudent(module_trait_cor, nrow(trait_data))

# Extract correlation and p-value for Green module vs Dengue

correlation_value <- module_trait_cor["green", "Infected with Dengue"]

p_value <- module_trait_pval["green", "Infected with Dengue"]

cat("Correlation with Dengue (Green module):", correlation_value, "\n")

cat("P-value:", p_value, "\n")

# ==== Step 4: Reactome Enrichment ====

if (abs(correlation_value) > 0.5 && p_value < 0.05) {

cat("Proceeding with Reactome enrichment...\n")

# ==== Step 4.1: Convert Gene IDs to Entrez ====

entrez_ids <- tryCatch({

bitr(green_genes,

fromType = "ENSEMBL", # Corrected here

toType = "ENTREZID",

OrgDb = org.Hs.eg.db)

}, error = function(e) {

stop("ID conversion failed. Check gene IDs and OrgDb.")

})

# ==== Step 4.2: Reactome Enrichment Analysis ====

reactome_result <- enrichPathway(

gene = entrez_ids$ENTREZID,

organism = "human",

pAdjustMethod = "BH", # Corrected

pvalueCutoff = 0.05, # Adjusted

qvalueCutoff = 0.05, # Adjusted

readable = TRUE

)

# ==== Step 4.3: Save Enrichment Results based on p.adjust ====

reactome_df <- as.data.frame(reactome_result)

# Order by adjusted p-value

reactome_df <- reactome_df[order(reactome_df$p.adjust), ]

write.csv(reactome_df, "Reactome_Enrichment.csv", row.names = FALSE)

**print(head(reactome_df))**

**Part2**

# ==== Load Required Libraries ====

library(ggplot2)

library(dplyr)

# ==== Step 1: Manually create dataframe for selected Reactome terms ====

reactome_selected <- data.frame(

Description = c(

"Influenza Infection",

"RHO GTPase Effectors",

"Viral mRNA Translation",

"Influenza Viral RNA Transcription and Replication",

"RHO GTPase cycle",

"SARS-CoV Infections",

"Apoptosis induced DNA fragmentation",

"MHC class II antigen presentation",

"Diseases of programmed cell death",

"SARS-CoV-2 Infection",

"Antiviral mechanism by IFN-stimulated genes",

"FCGR3A-mediated phagocytosis",

"Regulation of actin dynamics for phagocytic cup formation"

),

GeneRatio = c(

"35/622","49/622","23/622","27/622","55/622",

"51/622","6/622","19/622","17/622","33/622",

"19/622","9/622","9/622"

),

p.adjust = c(

1.396314e-09, 2.679636e-08, 4.846361e-08, 4.549969e-07, 1.682843e-06,

3.726182e-06, 4.894745e-04, 6.441214e-04, 7.426574e-04, 1.623956e-03,

5.468091e-03, 2.837875e-02, 3.387263e-02

)

)

# ==== Step 2: Convert GeneRatio to numeric ====

reactome_selected$GeneRatio <- sapply(strsplit(as.character(reactome_selected$GeneRatio), "/"),

function(x) as.numeric(x[1])/as.numeric(x[2]))

# ==== Step 3: Order pathways by adjusted p-value ====

reactome_selected <- reactome_selected %>%

arrange(p.adjust)

reactome_selected$Description <- factor(reactome_selected$Description,

levels = rev(reactome_selected$Description))

# ==== Step 4: Draw barplot (GeneRatio vs Reactome pathway, color by -log10(p.adjust)) ====

png("Reactome_Selected_Barplot.png", width = 10, height = 8, units = "in", res = 1500)

ggplot(reactome_selected, aes(x = GeneRatio, y = Description)) +

geom_bar(stat = "identity", aes(fill = -log10(p.adjust)), width = 0.6) +

scale_fill_gradient(low = "blue", high = "red", name = "-log10(adj p-value)") +

labs(title = "Reactome Pathways – Turquoise Module (Infected with DENV2): Antiviral and Immune Response",

x = "Gene Ratio",

y = "Reactome Pathway Description") +

theme_bw() +

theme(

axis.text.y = element_text(size = 10),

plot.title = element_text(hjust = 0.5, size = 14, face = "bold")

)

dev.off()
