# Supplementary material for: Comparative Gene Co-expression Network Analysis of Proviral and Antiviral Responses to Dengue Virus-2 (DENV-2) and Zika Virus (ZIKV) Infection in Human Neural Progenitor Cells (hNPCs)
Source: PLoS One. 2026 Apr 30;21(4):e0347540. doi: 10.1371/journal.pone.0347540 (PMC13132211; doi:10.1371/journal.pone.0347540)
Supplement: Attachment — Submitted filename: Response to Reviewers.docx [file pone.0347540.s011.docx]

15/03/2026

To

Academic Editor

PLOS One

Sub: Response to the Reviewers and Editor’s comments for the Reference [PONE-D-25-55299] - [EMID:b1d4d5d8cd1d0be5]

We sincerely thank the editor and reviewers for their careful reading and valuable suggestions regarding our manuscript. Following your guidance, we have slightly revised the manuscript title. The title is now:

“Comparative Gene Co-Expression Network Analysis of Proviral and Antiviral Responses to Dengue Virus-2 (DENV-2) and Zika Virus (ZIKV) in Human Neural Progenitor Cells (hNPCs)”. Previously, the title was: “Comparative Gene Co-Expression Network Analysis of Antiviral and Immune Responses to Dengue Virus-2 (DENV-2) and Zika Virus (ZIKV)”. We revised it to reflect the comparative analysis of proviral and antiviral responses of both viruses in hNPCs, which is the new focus of our study. This change did not involve major modifications to the manuscript content, as well as not changing in result or figure or tables; we only rearranged certain sections and added a few clarifying lines. All new additions and changes in the manuscript-reflecting the revised focus or responses to reviewer suggestions- are highlighted in yellow. Specifically, the titles of the Abstract and Conclusion sections are highlighted in yellow because the entire paragraphs were revised, while in the Methods, Discussion and References sections, only the individual lines or several references that were newly added are marked in yellow. Here, we have added eight new citations (6, 10, 51, 52, 53, 57, 61, 65), which are highlighted in yellow in the reference section. Additionally, five citations have been retracted or removed and are highlighted in red. We kindly request that the red-marked citations be removed from the reference section after your review.

Additionally, we performed minor edits throughout the whole manuscript to improve grammar, word choice, and formatting without altering the scientific content. These changes enhance readability and ensure consistency across the manuscript. We also have provided all responses to the Editor, Reviewer 1, and Reviewer 2, along with the revised manuscript, response letter, corrected figures, and Supporting Information files.

We hope that these revisions fully address the editorial and reviewer comments and improve the clarity, accuracy, and overall quality of our work. Finally, we have provided all responses to Editor, Reviewer 1, and Reviewer 2. In addition, we have included the revised manuscript, the response letter, all corrected figures, and the Supporting Information files. We believe these materials fully address the reviewers’ and editor’s comments and ensure that the manuscript meets the journal’s standards.

**Response to Editor’s comment**

**Response 1**

We have carefully reviewed the manuscript to ensure full compliance with PLOS ONE’s style requirements, including formatting, structure, and file naming conventions. All necessary revisions have been made to align with the journal’s guidelines and template.

**Response 2**

We thank the reviewers for pointing this out. We have carefully reviewed the manuscript and rephrased all overlapping text outside the Methods section. All relevant sources, including our previous publications, have been appropriately cited. These changes ensure that the manuscript now meets the journal’s standards for originality and proper attribution.

**Response 3**

We thank the editor for highlighting the code-sharing policy. All author-generated scripts used in this study have now been deposited in the supplementary file (S1_Code.docx) to get them permanently available upon publication.

**Response 4**

Raw Data Availability Statement

Raw RNA-seq data analyzed in this study are publicly available in the NCBI Sequence Read Archive (SRA) under BioProject PRJNA854905.

All data required to replicate the results of this study (including WGCNA-related graphs, values, and figures) are provided within the manuscript and its Supporting Information files. The following files are included:

- S1 Table:
- S1_Data_Set_metadata.csv, S2_Data_Set_vst_
- S2_Table: (a, b), S3_Table: (a, b) S4_Table: (a, b)

These files constitute the minimal data set required to replicate all study findings, including raw counts, metadata, processed matrices, and functional enrichment results. All Supporting Information files will be permanently available upon publication.

**Response 5**

We thank the editor for pointing this out. The supplementary files have now been re-uploaded with the correct file type as “Supporting Information.” In addition, legends for each Supporting Information file have been added to the manuscript after the References section, as requested.

**Response 6**

We confirm that no specific additional citations were recommended by the reviewers. All references included in the revised manuscript have been carefully selected based on their relevance to the study.

**Response 7**

We have carefully reviewed the entire reference list to ensure accuracy, completeness, and consistency with the journal guidelines. Seven new citations (10, 51, 52, 53, 57, 61, 65) have been added and are marked in yellow in the reference section. Additionally, five citations have been retracted/removed and are highlighted in red in the reference section.

**Response to Reviewer’s comments**

**Reviewer 1**

**i) Method**

We have revised the Materials and Methods section to clarify the criteria used for RNA-seq dataset selection. The dataset was selected based on biological relevance (human neural progenitor cells), inclusion of both DENV-2 and ZIKV infections under comparable conditions, consistent infection time point (72 h post infection), balanced sample representation, and sufficient sample size to support robust WGCNA analysis. Additional details regarding infection conditions and period have now been included in the S1 Table: SRA accessions of RNA sequences.

**ii) Discussion**

In the revised manuscript, we have added a paragraph in the Discussion section addressing the neurotropism of Zika virus that is marked as yellow in color in the revised manuscript.

**Reviewer 2**

We clarify that the RNA-seq data (BioProject PRJNA854905, GEO: GSE207347) were previously generated and published by Singh K, Martinez MG, Lin J, Gregory J, Nguyen TU, Abdelaal R, Kang K, Brennand K, Grünweller A, Ouyang Z, Phatnani H. Transcriptional and translational dynamics of Zika and dengue virus infection. Viruses. 2022 Jun 28; 14(7):1418. DOI: [https://doi.org/10.3390/](https://doi.org/10.3390/v14071418)]  that is cited as [21] in the introduction part of this revised manuscript. The author conducted transcriptional and translational dynamics of DENV2- and ZIKV-infected human neural progenitor cells were analyzed. However, they did not investigate higher-order gene co-expression patterns or identify regulatory hub genes within infection-associated modules. Our study represents the first application of weighted gene co-expression network analysis (WGCNA) to these publicly available RNA-seq data from NCBI, allowing us to uncover network-level host responses and hub genes specific to DENV2 and ZIKV infection in neural progenitor cells. This statement is now clearly given in the last part of the introduction part.

**Best Regards**

**Md. Ahashan Habib Siam**

**University of Chittagong, Bangladesh**
